# Soft Robotic Engines with Non‐Reciprocal Motion by Physical Intelligence

**DOI:** 10.1002/adma.202511630

**Published:** 2025-09-01

**Authors:** Oliver Skarsetz, Piet J.M. Swinkels, Jacqueline Figueiredo da Silva, Giulia Vozzolo, Marcos Masukawa, Giorgio Fusi, Brigitta Dúzs, Yanis Lassiat, Christoph Drees, Viacheslav Slesarenko, Andreas Walther

**Affiliations:** ^1^ Life‐Like Materials and Systems, Department of Chemistry University of Mainz Duesbergweg 10‐14 55128 Mainz Germany; ^2^ Max Planck Institute for Polymer Research Ackermannweg 10 55128 Mainz Germany; ^3^ POLYMAT Joxe Mari Korta Center University of the Basque Country UPV/EHU Avda. Tolosa 72 Donostia–San Sebastián 20018 Spain; ^4^ Cluster of Excellence livMatS @ FIT—Freiburg Center for Interactive Materials and Bioinspired Technologies University of Freiburg Georges‐Köhler‐Allee 105 79110 Freiburg im Breisgau Germany

**Keywords:** adaptive materials, embodied intelligence, hydrogels, intelligent matter, soft robotics

## Abstract

Movement is essential for living systems, enabling access to food, habitats, or escape from threats. Across scales, a key unifying principle is symmetry breaking to achieve non‐reciprocal motion and accumulate work. In soft robotics, many actuators mimic biological responsiveness, but they typically exhibit reciprocal motion, where forward work is canceled in the return stroke – preventing work accumulation in cyclic operation. Here, a simple and broadly applicable hydrogel engine concept is presented that overcomes this limitation by encoding kinetic asymmetry into swelling and deswelling transitions. This hard‐coded asymmetry yields non‐reciprocal motion trajectories, enabling continuous mechanical work extraction under a single, uniform stimulus – without complex external control. The strategy embodies a material‐based ratchet mechanism rooted in physical intelligence, independent of geometry or scale, and generalizable across stimuli. This hydrogel engine is implemented in soft robotic systems, including artificial cilia for fluid pumping and conveyor belts for object transport. Starting from macroscopic thermoresponsive systems, the design is extended to microscale formats via 3D printing and to other stimuli‐responsive materials. This approach shifts the paradigm in soft robotics – from increasing chemical complexity to leveraging intrinsic material properties for emergent function – paving the way for scalable, autonomous systems driven by physical intelligence.

## Graphical abstract

Movement is vital, and it requires breaking symmetry. This study introduces a hydrogel engine that achieves non‐reciprocal motion through kinetic asymmetry in repeated actuation cycles. This approach for breaststroke‐swimming‐like motion is embedded inside the material and establishes a new paradigm of physical intelligence for soft robotics.

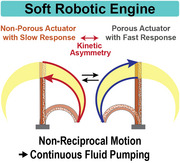

## 1 Introduction

Non‐reciprocal motion trajectories of biological motors or motor appendages are key to achieving locomotion and transport in the microscopic world, such as in flagella for bacterial movement^[^ ^1^, ^2^ ^]^ or for cilia arrays that establish unidirectional fluid flow along cell surfaces.^[^ ^3^, ^4^ ^]^ Our macroscale analogue to non‐reciprocal motion is breaststroke swimming.^[^ ^5^ ^]^ Non‐reciprocal motion principles are mostly hard‐coded in structures and kinetic asymmetry. Due to their small size, flagella and cilia operate at low Reynolds numbers, in which reciprocal actuation cannot achieve net movement as forward and backward motion cancel each other.^[^ ^6^, ^7^ ^]^ “Motion” is defined with an elementary actuation trajectory, which can be either reciprocal or non‐reciprocal. “Movement” refers to the net displacement observed after multiple motion cycles. Movement accumulates during cycles of non‐reciprocal motion (see Table S1, Supporting Information).

The burgeoning field of synthetic soft robotics has yielded a rich variety of actuators that respond to external stimuli with motion – most notably through bilayer actuators. Many different components, such as responsive hydrogels,^[^ ^8^, ^9^, ^10^, ^11^, ^12^ ^]^ shape memory polymers,^[^ ^13^, ^14^, ^15^ ^]^ dielectric actuators,^[^ ^16^, ^17^ ^]^ or liquid crystal elastomers^[^ ^18^, ^19^, ^20^ ^]^ have been developed and incorporated into different configurations to achieve varying kinematic motion.^[^ ^21^ ^]^ Although complex motion paths can be programmed to suit different applications, soft robotic devices suffer from a key limitation in contrast to biological systems: they typically generate reciprocal motion trajectories during cyclic operation.^[^ ^22^ ^]^ As a result, no net work or movement is accumulated. Any work performed in one direction is negated when the device returns to its starting position. Overcoming this challenge by developing concepts that transition from reciprocal to non‐reciprocal motion in cyclic operations remains a significant hurdle. Addressing this could greatly expand the field of actuators, moving closer to the creation of true soft machines.^[^ ^23^ ^]^

To accumulate work in cyclic operations of soft robots, it is necessary to introduce time‐reversal asymmetry into the motion trajectories.^[^ ^7^ ^]^ Current approaches rely on complicated external control mechanisms operating at different actuation stages or require sophisticated multicomponent designs. For instance, temporally and spatially controlled light stimulation,^[^ ^24^ ^]^ sequential application of orthogonal stimuli in non‐reciprocal order,^[^ ^25^, ^26^ ^]^ self‐shadowing under laser irradiation,^[^ ^27^ ^]^ or combining photoswitches with different kinetics^[^ ^28^ ^]^ have been suggested. Additionally, reciprocal motion can be biased in one direction using an externally provided, ratcheted surface or through friction variation.^[^ ^29^, ^30^, ^31^, ^32^ ^]^ Critically, in all these examples of kinetic asymmetry in stimulus and interaction with the environment, the non‐reciprocal trajectory is either highly sensitive to experimental arrangement or requires externally imposed timing with computer control to guide the stimulus application. This is a complicated process for applications, and it would be hard to ensure the autonomous orchestration of multiple stimuli. Non‐reciprocal motion has been achieved by incorporating holes to spatially enhance swelling into 3D printed geometries. However, this concept cannot be generalized to more complex soft robotic machines, and such holes compromise the structure.^[^ ^33^ ^]^

We propose a radically simple and universally applicable design paradigm to transform reciprocal‐motion soft robotic actuators into non‐reciprocal‐motion soft robotic engines. By simply engineering the swelling and deswelling kinetics of hydrogel actuators through controlled porosity, our design enables these engines to continuously convert uniformly applied stimulus and counter‐stimulus cycles into extractable work and movement. This approach embeds material intelligence physically,^[^ ^34^ ^]^ making chemical complexities and external controls largely redundant. Indeed, we generalize the concept to different geometries, stimuli, and length scales. First, we fabricate artificial cilia for continuous fluid propulsion and a conveyor belt for macroscopic object transport. Second, we generalize the stimulus from thermal switching to pH switching. Third, the soft robotic engines are downscaled with microscale continuous optical printing, which allows the simultaneous fabrication of cilia arrays. Particle image velocimetry of fluorescent beads visualizes and quantifies the effective fluid pumping of the actuating cilia arrays. Finally, finite element simulations were employed to navigate the vast parameter space and optimize both kinetic asymmetry and the geometries of the soft robotic engines, maximizing the accumulated work.

## 2 Results and Discussion

### 2.1 Conceptual Design of Soft Robotic Engines

**Figure 1:**
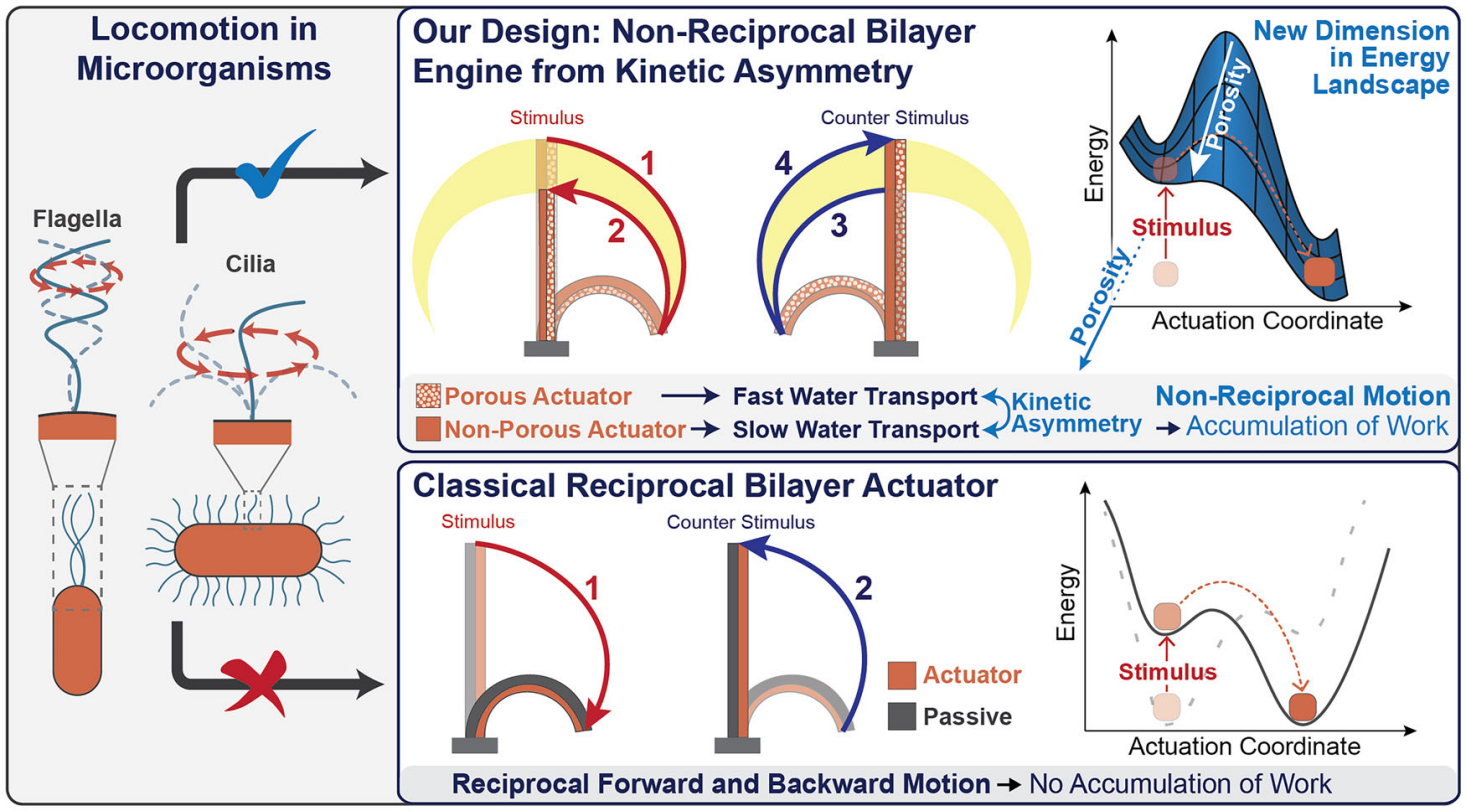
Soft robotic engines with kinetic asymmetry can accumulate work via non‐reciprocal trajectories, whereas classical bilayer actuators show reciprocal motion without accumulation of work. Our soft robotic engine (a cilium) consists of actuating materials with different kinetics and shows a resulting non‐reciprocal actuation trajectory. Work is accumulated in a cyclic operation. On the other hand, the classical bilayer actuator consists of a passive and a responsive actuator material, where the forward and backward motions are identical. Under cyclic operation, no work is accumulated.

**Figure** **1** contrasts our design paradigm for hydrogel engines with established classical hydrogel bilayer actuators that dominate the field of hydrogel soft robotics.^[^ ^21^, ^35^, ^36^ ^]^ Classical bilayers consist of one passive and one responsive actuator material (Figure 1, bottom). In these devices, the motion is reciprocal—both forward and backward motions are identical during the application of stimulus and counter‐stimulus, resulting in no net work accumulation during cyclic operation. We hypothesized that non‐reciprocal trajectories and work accumulation could be achievable in such devices if we were able to implement kinetic asymmetry into the swelling and deswelling transitions, allowing one side to contract and swell faster than the other side. We envisaged that this is even possible for chemically identical responsive materials on both sides, thus by hard‐coding the kinetic asymmetry as an exclusive form of physical intelligence into the structure of the materials (Figure 1, top; Movie S1, Supporting Information).

The critical point to achieve kinetic asymmetry is to encode different porosities into hydrogel bilayers, where both sides consist of the same actuating material (Figure 1, top). Porosity enhances water transport in and out of the hydrogel, thus accelerating swelling and deswelling kinetics.^[^ ^37^, ^38^ ^]^ During stimulus application, the porous hydrogel (right side) contracts faster than the non‐porous one (left side), which results in a transient bilayer bending to the right, followed by straightening with a shortened length due to the deswelling of the left hydrogel side. Applying a counter‐stimulus causes a temporary leftward bend due to faster swelling of the right, porous layer, followed by a return to the original straight position. This results in the soft robotic engine following a distinct non‐reciprocal trajectory through defined non‐equilibrium states, functioning similarly to a cilium and accumulating work in cyclic operation. It solely uses one globally applied stimulus/counter‐stimulus pair without the need for complex spatially or temporally orchestrated stimuli, because the stimulus‐information‐processing is hard‐coded on a structural level.

### 2.2 Artificial Cilium from Built‐In Kinetic Asymmetry

**Figure 2:**
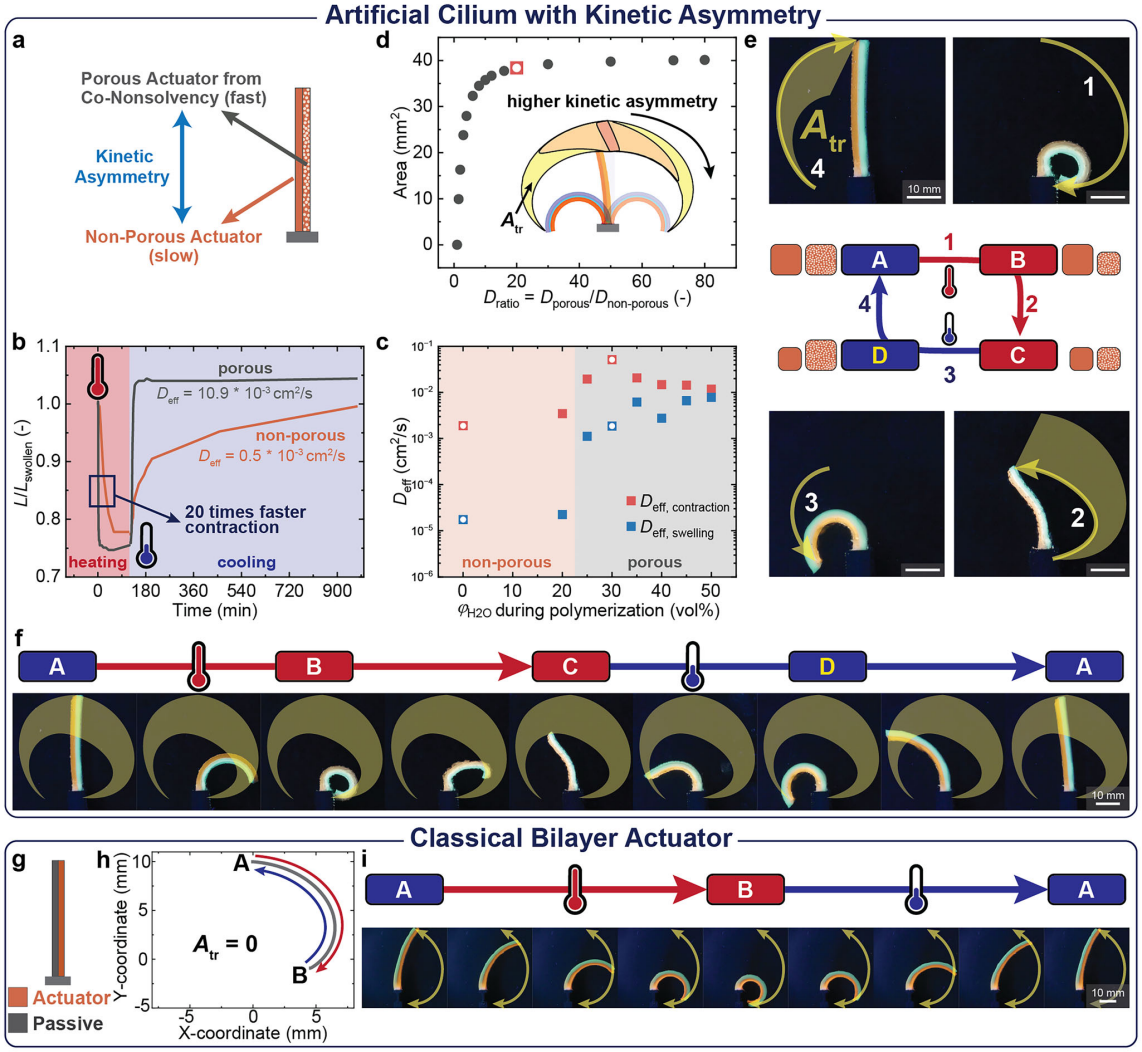
The first soft robotic engine with bilayer geometry mimics an artificial cilium that pumps liquid unidirectionally. a) The bilayer consists of one porous layer with fast actuation and one non‐porous layer with slow actuation. Porosity is introduced with co‐nonsolvency. b) Kinetic asymmetry in the actuation of the materials: Contraction of individual, equilibrium‐swollen hydrogel rods (*L* × *w* × *h* = 26 × 5.2 × 5.2 mm) during heating to 60 °C by plotting *L*/*L_swollen_* over time. After 2 h, the high temperature stimulus is removed and the hydrogels re‐swell. c) Experimentally determined *D_eff_* for contraction and swelling for hydrogels synthesized from different DMSO/water mixtures. The DMSO/water mixtures *φ*_*H*2*O*_ = 30% and *φ*_*H*2*O*_ = 0% (indicated with white squares) are selected as porous and non‐porous materials due to the greatest ratio *D_ratio_* = *D*_*eff*,*porous*_/*D*_*eff*,*non*−*porous*_. d) FE simulations predict the *A_tr_* of the bilayer tip for different *D_ratio_*: *A_tr_* increases for *D_ratio_* < 10 and then levels off. The experimentally employed *D_ratio_* is marked in red. e) Non‐reciprocal actuation during one actuation cycle follows four distinct actuation states. f) Full actuation trajectory over one heating/cooling cycle: Time‐reversal symmetry of the actuation is broken. The *A_tr_* traced by the bilayer tip quantifies the accumulated work. g) In contrast to the soft robotic engine, the classical bilayer consists of one active and one passive material. h) FE simulation reveals the identical trajectory of the bilayer tip, where the *A_tr_* is zero. i) The active layer contracts during heating and recovers with an identical trajectory during cooling. The full actuation trajectory over one heating/cooling cycle is symmetric in time.

We target poly(*N*‐isopropylacrylamide) (pNIPAAm) hydrogels as the first material to build soft robotic engines (**Figure** **2**a). Crosslinked pNIPAAm forms robust thermo‐responsive hydrogels, known to 1) undergo swelling/deswelling transitions at around 32 °C and 2) exhibit co‐nonsolvency during polymerization in solvent mixtures, allowing to engineer controlled porosity. Non‐porous gels result from polymerizing NIPAAm in dimethyl sulfoxide (DMSO) or water, whereas mesoporous structures form by phase segregation when polymerized in DMSO/water mixtures.^[^ ^38^, ^39^, ^40^ ^]^ Superresolution imaging by structured illumination microscopy visualizes the porosity (Figure S1, Supporting Information).

Figure 2b depicts an exemplary comparison of non‐porous and porous pNIPAAm hydrogels obtained from polymerizations in pure DMSO and a DMSO/water mixture (*φ*_H2O_ = 30%). Upon immersion of fully swollen hydrogel rods (length *L* × width *w* × height *h* = 26 × 5.2 × 5.2 mm) into a 60 °C water bath, the porous hydrogel contracts within minutes while the non‐porous hydrogel requires over 1 h. The total contraction *L*/*L*_swollen_ is similar. Upon cooling to room temperature, the porous hydrogel re‐swells 12 times faster than the non‐porous one. This underscores the basic elements of kinetic asymmetry – porous gels contract and swell faster!

The Tanaka‐Fillmore theory allows for quantifying the effect of porosity on polymer networks by determining the effective diffusion coefficient *D*_eff_ from swelling experiments, which corresponds to the speed of water transport (Figure 2c).^[^ ^41^, ^42^ ^]^ For hydrogels prepared at water contents *φ*_H2O_ > 20%, the *D*_eff_ increases by more than one order of magnitude, signifying substantial porosity. *D*_eff_ values are consistently lower during the re‐swelling compared to contraction. This is due to slower water diffusion into the collapsed hydrogel, which requires additional wetting of the polymer chains.^[^ ^43^ ^]^ We introduce the parameter *D*_ratio_ = *D*_eff,porous_/*D*_eff,non−porous_ to quantify the kinetic asymmetry for devices combining two hydrogels with different *D*_eff_.

To understand the parameters influencing the extent of non‐reciprocal motion, we conducted finite element (FE) simulations of a bilayer engine geometry (Figure 2d), using experimental parameters as input (see supplementary text). By conducting parametric sweeps and tracking the motion trajectory via the XY position of the bilayer tip, we derived the area of the trajectory (*A*_tr_) of the non‐reciprocal motion (Figure S2a, Supporting Information). This parameter also semi‐quantitatively estimates the magnitude of fluid transport (i.e., the work done) exerted by such a cilium on its surroundings. The simulations reveal key tendencies:

- Higher *D*_ratio_ increase *A*_tr_ initially (Movie S2, Supporting Information), but the effect plateaus at *D*_ratio_ ≈ 20. Beyond this point, the porous hydrogel completes the actuation before the non‐porous even begins. Such *D*_ratio_ are accessible by our porosity engineering (the experimental system shown in Figure 2b is indicated in Figure 2d with a red dot).
- Similar stiffness for the two actuating layers (*E*_ratio_ = *E*_porous_/*E*_non−porous_ close to 1) maximizes *A*_tr_ (Figure S2b, Supporting Information).
- Increasing the aspect ratio (height to width) of the bilayer engine enhances *A*_tr_ (Figure S2c, Supporting Information).
- *A*_tr_ increases with increasing actuation strain (i.e., higher contraction), which is an intrinsic material property and therefore challenging to vary experimentally (Figure S2d, Supporting Information).

Based on these insights from FE simulations, and considering accessible material parameters, we selected pNIPAAm hydrogels with *D*_ratio_ = 22 (*φ*_H2O_ = 30% and *φ*_H2O_ = 0%), *E*_ratio_ ≈ 0.6, and an actuation strain of 38% (Figure S3, Supporting Information). Although the hydrogels have the same polymer content, the stiffness of the porous hydrogel is 43% lower than the stiffness of the non‐porous hydrogel (Figure S4, Supporting Information).

We then built the first soft robotic engine using a bilayer geometry (*L* × *w* × *h* = 30 × 3 × 4 mm). The actuation during one cycle of stimulus/counter‐stimulus application indeed follows the proposed four actuation states (Figure 2e,f and Movie S3, Supporting Information). Initially, at room temperature, both active pNIPAAm layers are swollen, and the bilayer is straight (state A). Upon stimulus application (heating to 60 °C), the bilayer transiently bends to the right (state B) because of the faster contraction of the porous pNIPAAm layer. This represents a non‐equilibrium state, where the porous structure alone is in equilibrium contraction due to the faster deswelling kinetics. Over time, the non‐porous pNIPAAm also contracts, bringing the bilayer to a straight but contracted equilibrium position (state C). Upon counter‐stimulus application (cooling to room temperature), the porous pNIPAAm layer expands faster, causing bending to the left (state D). Eventually, the non‐porous pNIPAAm also expands, realigning the bilayer to its initial position (state A). The experimental area traced by the tip (*A*_tr,exp_; yellow area in Figure 2e) reaches 540 mm^2^, which is comparable to the simulated *A*_tr,sim_ of 710 mm^2^.

Next, we emphasize the uniqueness of our design by comparing it to state‐of‐the‐art bilayer actuators. First, we consider a classical bilayer configuration consisting of one passive, non‐responsive layer and one active layer (Figure 2g; Movie S3, Supporting Information). FE simulations show purely reciprocal actuation (Figure 2h). The bending trajectory is identical forward and backward, resulting in *A*_tr_ = 0. Experimentally, using poly(acrylamide) (pAAm) as passive and pNIPAAm as active material, the bilayer bends into its equilibrium state upon heating to 60 °C (Figure 2i). When cooled to room temperature, the active layer re‐swells, and the bilayer returns to its initial state. Forward and backward motions are identical, and no metastable, non‐equilibrium states are observed. Extending our comparison to a multiresponsive bilayer actuator (Figure S5, Supporting Information), we constructed a bilayer combining a pNIPAAm hydrogel with a volume phase transition temperature (VPTT) of ≈32 °C with a p(NIPAAm‐*co*‐AAm) hydrogel with a higher VPTT of ≈42 °C.^[^ ^44^ ^]^ Although this combination leads to three actuation states, it also lacks time‐reversal asymmetry in the motion trajectory, and the forward and backward motions remain identical. Both controls thus highlight the critical effect of engineering swelling/deswelling kinetic asymmetry as physical intelligence into bilayers to generate cilium‐like bilayer engines.

### 2.3 Macroscale Object Transport and Microscale Liquid Transport

**Figure 3:**
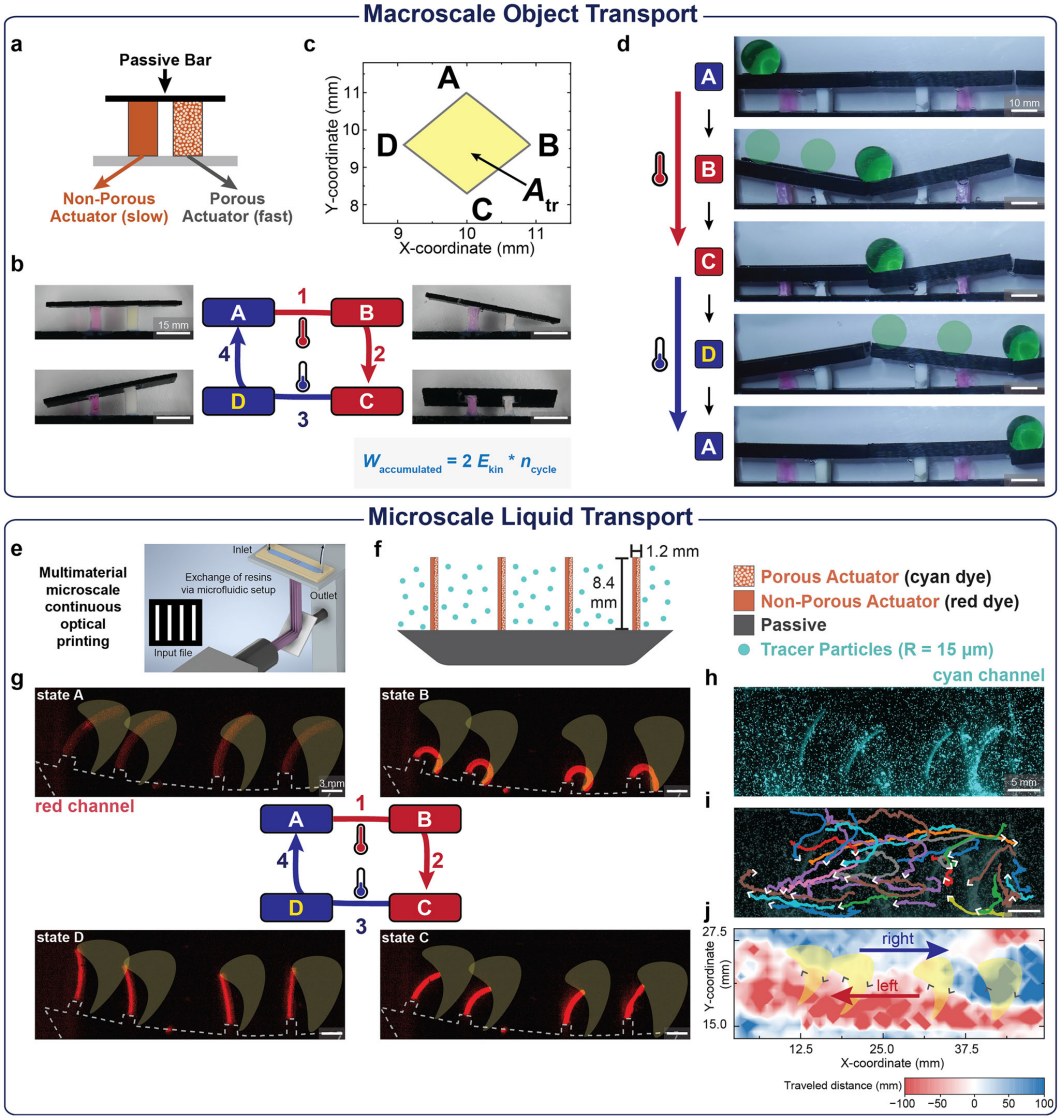
Macroscale object transport and microscale liquid transport. a) The second soft robotic engine with seesaw geometry can be arrayed into a conveyor belt to continuously move macroscopic objects. The seesaw consists of a movable non‐hydrogel passive bar that is connected to two active materials. The same porous and non‐porous hydrogels as in the first soft robotic engine are used. To facilitate imaging, the non‐porous hydrogel contains rhodamine B acrylate, and the porous hydrogel contains fluorescein acrylate. b) The non‐reciprocal actuation is described in four actuation states. c) FE simulation reveals the non‐reciprocal trajectory and resulting *A_tr_* of the tracked center of the seesaw. d) An array of seesaw engines that are alternatingly flipped transports a sphere twice during one actuation cycle. Work accumulates in each cycle. e) µCOP inside microfluidic polydimethylsiloxane (PDMS) chips allows the sequential photopolymerization with ink exchange to obtain multimaterial hydrogel structures. f) The cilia array consists of four bilayers that are simultaneously printed and combined with a passive baseplate. Tracer particles are added to visualize the fluid flow. g) Fluorescent images (*λ*_*excitation*_ = 640 nm) of the four actuation states of the rhodamine B‐containing cilia array. h) Fluorescent particles (*λ*_*excitation*_ = 555 nm, *ρ* = 1.0 g mL^−1^) are suspended in water to allow tracing of the liquid flow via PIV. i) Traces of selected individual particles reveal the net displacement in x‐ and y‐direction. j) PIV analysis of individual particles, where the net flow in x‐direction is calculated at each coordinate. Regions, where particles on average move toward the right, are marked in blue, while red marks regions of net movement to the left. The *A_tr_*, which is traced by the artificial cilia, is colored in yellow.

After successfully demonstrating artificial cilia based on a bilayer engine, we extended our design to a second soft robotic engine featuring a seesaw geometry, in which a horizontal bar is connected to two hydrogel supports with kinetic asymmetry (**Figure** **3**a). Despite its simplicity, this configuration achieves a unique non‐reciprocal trajectory. The actuation follows four states with transient tilting to the right and left during one cycle of stimulus/counter‐stimulus application (Figure 3b). For a *D*_ratio_ = 22, the movement of the seesaw center results in an experimental non‐reciprocal trajectory area of *A*_tr,exp_ of 2.5 mm^2^, which is comparable to the simulated *A*_tr,sim_ of 3.4 mm^2^ (Figure 3c). In contrast, a classical seesaw actuator with passive and active material actuates reciprocally, with a resulting *A*_tr_ = 0 (Figure S6, Supporting Information).

To showcase work accumulation in a macroscopic context, we arrayed seesaw engines with alternating orientations into a conveyor belt and placed a sphere on top (Figure 3d; Figure S7, Supporting Information). During operation, the seesaws tilt appropriately, and the marble rolls from its initial position to the second seesaw. As time progresses, the seesaws straighten to the lower equilibrium position at 60 °C. Applying the counter‐stimulus (cooling to room temperature) leads to further non‐reciprocal tilting, which triggers the sphere to roll to the third seesaw. With each cycle, the sphere moves twice, hence the work of *W*_cycle_ = 2*E_kin_ is accumulated. The sphere moves from one to the next seesaw due to inertia.

Furthermore, the seesaw configuration reiterates the vital role of kinetic asymmetry. Even employing two thermoresponsive actuators with different VPTTs in a multiresponsive setup does not yield non‐reciprocal motion or work accumulation (Figure S8a–c, Supporting Information). An array of alternatingly oriented multiresponsive seesaw actuators can only move objects a single step at most (Figure S8d, Supporting Information).

After developing an artificial cilium and a conveyor belt, we further advanced our technology to create more complex geometries without manual assembly and to achieve faster actuation speeds at smaller scales through the use of microscale continuous optical printing (µCOP) (Figure 3e; Figure S9, Supporting Information). µCOP is a multimaterial manufacturing technique that uses image projection to locally photopolymerize hydrogel resins within microfluidic chips.^[^ ^45^ ^]^ We printed an array of bilayer cilia onto a passive baseplate via sequential photopolymerization with intermittent resin exchange (Figure 3f). After printing, we transferred the cilia array into a water‐filled aluminum channel (Figure 3g; Figure S10, Supporting Information).

Similar to the cm‐scale artificial cilium (Figure 2e), the mm‐scale µCOP cilia array actuates in four actuation states with transient bending to the right during heating and transient bending to the left during cooling (red channel; Figure 3g; Movie S4, Supporting Information). Importantly, the total actuation time is reduced by a factor of 4 as the size of the actuators is halved.^[^ ^41^ ^]^ To verify if this non‐reciprocal actuation translates to fluid displacement, we employed particle image velocimetry (PIV) using fluorescent particles that can be imaged simultaneously with the labelled bilayer actuator (cyan channel; Figure 3h). We traced the position of each fluorescent particle over time to determine their displacement in a field of view of 46 × 29 mm (Figure 3i). Furthermore, we subdivided into areas of 1 × 1 mm and tracked the x‐displacement of all particles present in that area to determine whether particles in a region travel to the left (negative values, red color) or to the right (positive values, blue color) during one actuation cycle (Figure 3j). The non‐reciprocal actuation of the cilia array results in a distinct particle movement pattern. Particles in the upper Y‐coordinates predominantly move to the right, while those in the lower Y‐coordinates move to the left. Averaging these displacements over time reveals a net flux of 90 µm min^−1^ toward the left. Furthermore, the actuation of the cilia is consistent over multiple actuation cycles, with a sweeping area of 15 ± 3 mm^2^ over 17 heating/cooling cycles (Figure S11, Supporting Information). Overall this cilia array gives first insights into the applicability for autonomous microfluidic transport systems.

To explore the generalizability of the kinetic asymmetry concept, we further expanded our system design from thermal to pH response as one important example. By incorporating acrylic acid (AA) into our pNIPAAm systems, we developed acid‐responsive elements for both porous and non‐porous hydrogels. Isothermal swelling tests following pH changes demonstrate that the porous hydrogel exhibits significantly faster contraction and swelling with a *D*_ratio_ = 13 (Figure S12a, Supporting Information). To validate the kinetic asymmetry in a soft robotic engine, we further fabricated a pH‐responsive porous/non‐porous p(NIPAAm‐*co*‐AA) bilayer device. During a pH actuation cycle, the pH bilayer engine also accesses four actuation states with a non‐reciprocal trajectory, thereby validating that our concept can be effectively generalized to different stimuli (Figure S12b, Supporting Information).

## 3 Conclusion

We have introduced a potentially transformative engineering paradigm in soft robotics, converting reciprocal soft robotic actuators into non‐reciprocal soft robotic engines capable of work accumulation—an ability previously unattainable in classical designs. Our approach leverages physical intelligence to encode kinetic asymmetry into swelling and deswelling transitions, effectively creating a material‐embodied ratchet mechanism. This fundamentally shifts how soft robotic motion is designed and controlled, even with the simplest unstructured stimulus, offering a scalable and chemically agnostic solution.

We demonstrated this concept with two soft robotic engine architectures—cilia and seesaw—fabricated through various printing methods and generalized the approach from thermal to isothermal operation. Our developments enabled the realization of a conveyor belt‐type soft machine for continuous object transport and artificial cilia arrays for fluid pumping, illustrating the practical applications of extractable work. Additionally, the simplicity of our design allows FE simulations to serve as a predictive tool, streamlining the optimization of kinetic asymmetry for different materials and geometries.

By embracing simplicity in material selection and harnessing intrinsic physical intelligence, we hope to inspire new directions in soft robotics that transcend limitations in stimulus, scale, and environmental constraints. Our approach should also be applicable to other stimulus‐responsive materials, such as light‐responsive spiropyran‐containing hydrogels. Furthermore, other pore‐forming strategies, such as gas foaming or particle leaching, could achieve a similar kinetic asymmetry to enable non‐reciprocal actuation. Unlike conventional approaches that rely on complex multi‐stimulus actuation, our strategy uses porosity engineering to achieve kinetic asymmetry in chemically identical materials, enhancing both scalability and real‐world applicability. This concept opens the door for broader technology transfer, including potential adaptation to dielectric elastomers,^[^ ^46^ ^]^ which could extend our approach to water‐free bulk soft robotic systems.

Ultimately, our findings challenge the field of soft robotics, which either advances toward higher chemical complexity or increasingly sophisticated externally controlled actuation strategies involving human or computer input. Instead, leveraging physically encoded intelligence allows for achieving new forms of autonomous motion and mechanical work accumulation through simple means. Looking ahead, the integration of our design principle with additional elements of physical intelligence, such as memory, adaptation, and feedback, is needed to bridge the gap between artificial machines and biological systems.^[^ ^47^ ^]^

## Conflict of Interest

The authors declare no conflict of interest.

## Author Contributions

O.S. and A.W. conceptualized the project. O.S. planned and performed the hydrogel synthesis and fabrication of soft robotic engines. O.S. built the microscale continuous optical printing setup. O.S. conducted the particle image velocimetry experiments, which were analyzed by P.S. P.S. wrote the Python code to control the image projection for printing and helped with the printing setup. J.F. contributed to the fabrication of advanced soft robotic engines. G.V. conducted small molecule synthesis. O.S. and M.M. developed the procedure to fabricate the microfluidic chips. G.F. helped with the synthesis of pH‐responsive hydrogels and the experimental setup. B.D. helped with the microscale fabrication technique and hydrogel synthesis. Y.L. worked on the microscale printing under the supervision of O.S. C.D. conducted the superresolution microscopy. O.S. and V.S. performed finite element simulations. O.S. wrote the draft, which was revised by B.D. and A.W.

## Supporting information



Supporting Information

Supplemental Movie 1

Supplemental Movie 2

Supplemental Movie 3

Supplemental Movie 4

## Data Availability

The data that support the findings of this study are available from the corresponding author upon reasonable request.
