# Supplementary material for: Soft Robotic Engines with Non‐Reciprocal Motion by Physical Intelligence
Source: Adv Mater. 2025 Sep 1;37(45):e11630. doi: 10.1002/adma.202511630 (PMC12617052; doi:10.1002/adma.202511630)
Supplement: Supplementary file 1 — Supporting Information [file ADMA-37-e11630-s003.pdf]

ADVANCED MATERIALS

Supporting Information

for *Adv. Mater.*, DOI 10.1002/adma.202511630

Soft Robotic Engines with Non-Reciprocal Motion by Physical Intelligence

Oliver Skarsetz, Piet J.M. Swinkels, Jacqueline Figueiredo da Silva, Giulia Vozzolo, Marcos Masukawa, Giorgio Fusi, Brigitta Dúzs, Yanis Lassiat, Christoph Drees, Viacheslav Slesarenko and Andreas Walther**

1 Supplementary Information

1.1 Methods

Materials

N-isopropyl acrylamide (NIPAAm; ≥98 %) and acrylamide (AAm; ≥98 %) were purchased from TCI. Methylene bis acrylamide (Bis-AAm; ≥98 %), acrylic acid (AA; ≥99 %), lithium phenyl-2,4,6,-trimethylbenzoylphosphinate (LAP ≥95 %), fluorescein *o*-acrylate 95 %, and 1*H*,1*H*,2*H*,2*H*-perfluorooctyltriethoxysilane 98 % were purchased from Sigma Aldrich. Dimethyl sulfoxide (DMSO; ≥99 %) was purchased from Thermo Scientific Chemicals. Acryloxyethyl thiocarbamoyl Rhodamine B was purchased from Polysciences, Inc.

AA was passed through a short column of acidic alumina prior to use to remove the inhibitor. NIPAAm was recrystallized once from *n*-hexane.

Preparation of Hydrogel Resins

Resins for porous and non-porous pNIPAAm hydrogels were adapted from Tokuyama et al[1]. Both porous and non-porous hydrogel resins were prepared by dissolving identical amounts of NIPAAm monomer with Bis-AAm cross-linker and LAP photoinitiator, where only the ratio of DMSO to water was varied. Resin for porous hydrogel: Dissolution of 2.5 M NIPAAm with 3.7 mol% Bis-AAm cross-linker and 3.4 mM LAP photoinitiator in 70/30 v/v% DMSO/water (φ_{H2O} = 30 %). Resin for non-porous hydrogel: Dissolution of 2.5 M NIPAAm with 3.7 mol% Bis-AAm cross-linker and 3.4 mM LAP photoinitiator in 100/0 v/v% DMSO/water (φ_{H2O} = 0 %). For systematic studies, hydrogels with further DMSO/water ratios from φ_{H2O} = 0 % to φ_{H2O} = 50 % were explored. Fluorescein *o*-acrylate was added to the porous hydrogel resin and acryloxyethyl thiocarbamoyl Rhodamine B was added to the non-porous hydrogel resin to enable fluorescence imaging. Resin for a passive (non-responsive) hydrogel: Dissolution of 2.5 M AAm with 3.7 mol% Bis-AAm cross-linker and 3.4 mM LAP photoinitiator in 100/0 v/v% DMSO/water (φ_{H2O} = 0 %).

Manufacturing of PTFE Molds

The geometries were photopolymerized in PTFE molds: 2D computer aided design models of hydrogel actuators for the desired kinetic studies, bilayers and seesaws were milled into PTFE blocks (length *L* × width *w* × height *h* = 100 × 60 × 10 mm) with varying depth using a computer numerical control machine with a 1 mm diameter milling tool.

Kinetic Studies to Determine the Effective Diffusion Coefficients

For the studies of swelling equilibria and swelling kinetics, rectangular hydrogel rods with aspect ratio 5 (*L* × *w* × *h* = 20 × 4 × 4 mm) were prepared by photopolymerization for 60 s with ultraviolet (UV) light (NailStar UV lamp 4 × 9 W bulbs, λ_{max} = 365 nm) in a PTFE mold. Afterwards, the hydrogels were carefully removed from the mold and placed in excess water to completely remove DMSO. When necessary, the solution was exchanged. Although the hydrogels contain different φ_{H2O} during preparation, all equilibrated hydrogels contain only water.

For actuation with high temperature, the hydrogel rods were heated in a round petri dish (*d* = 88 mm, with 100 mL water) on a hotplate set to 85 °C from below using a magnetic stirrer (IKA RCT digital). In this setup, the water reached a temperature of 60 °C. For re-swelling, ice was added to the water for fast cooling to room temperature. Images were recorded every 30 s. For actuation of multiresponsive actuators with two intermediate temperatures (Figure S5 and Figure S8), the water in the round petri

dish (d = 88 mm, with 100 mL water) was first heated to 40 °C and then heated to 60 °C in a second step. The actuation strain was calculated as $\varepsilon_{\mathrm{A}} = 1 - L_{\mathrm{contracted}}/L_{\mathrm{swollen}}$ (Figure S3).

For quantification of the swelling kinetics, the effective diffusion coefficients (D_{eff}) were calculated from the Tanaka-Fillmore model[2-3], by fitting equation E1 to obtain the relaxation time τ.

$$\frac{d(t)-d(t_{\infty})}{d_{\mathrm{i}}-d(t_{\infty})} \approx \frac{6}{\pi^2} \exp\left(-\frac{t}{\tau}\right) \qquad \text{E1}$$

From the relaxation time, the D_{eff} was calculated using equation E2.

$$D_{\mathrm{eff}} = \frac{d(t_{\infty})^2}{\pi^2 \tau} \qquad \text{E2}$$

Note that the D_{eff} were determined for the actuation of rectangular rods, hence, the values are 2/3 of the D_{eff} of a sphere[4].

Determination of the VPTT of the Hydrogels

To measure the VPTT, hydrogel rods were immersed in water and the temperature was increased with a hotplate (ColdPlate, qinstruments) controlled with a Python script from 22 °C to 68 °C with 2 °C increments every 20 minutes. The lengths of the hydrogels were measured at the end of each heating interval and divided with the length in dry state to obtain the swelling factor $SF_{\mathrm{L}} = L/L_{\mathrm{dry}}$.

Tensile Testing of the Hydrogels

To measure the stiffness, hydrogel dogbones (L × w × h = 20 × 4 × 2 mm) were photopolymerized, after which they were equilibrated in water. The stress–strain curves of the fully equilibrated hydrogels were measured under tensile deformation with a Shimadzu compact tabletop testing machine EZTest using a 5 N load cell and displacement rate of 1 mm s^{-1}. The hydrogel rods were superglued to paper that was clamped to reduce preliminary deformation of the hydrogel specimen. Porous pNIPAAm has a stiffness of E_{porous} = 45 kPa ± 3 kPa, while non-porous pNIPAAm has a stiffness of $E_{non-porous}$ = 64 kPa ± 4 kPa (n = 8).

Preparation and Actuation with Bilayer Geometry

The bilayer geometry was manufactured by sequential photopolymerization: First, a single layer (L × w × h = 30 × 4 × 1.5 mm) of the porous hydrogel precursor solution was polymerized for 60 s with UV light in a PTFE mold. Second, the precursor for the second non-porous layer (L × w × h = 30 × 4 × 1.5 mm) was filled into a second mold and polymerized for 60 s of UV light. Afterwards, the porous layer was placed on top of the non-porous layer and polymerized with a few drops of additional precursor to obtain interfacial polymerization. For imaging the bilayer with one constrained side, the bilayer was superglued to a fused filament fabrication (FFF) printed acrylonitrile butadiene styrene (ABS) block, which was further adhered with double sided tape (tesa® powerbond mirror) to a petri dish with black foil as background.

During actuation, the bilayer was imaged from above with a digital camera (Panasonic Lumix G70) under UV light (λ_{max} = 365 nm) in which both rhodamine B and fluorescein dye absorb to enhance contrast. Like before, the bilayer was actuated by heating the water to 60 °C. For re-swelling, ice was added to the water. An image was recorded every 30 s.

Control: A multiresponsive bilayer actuator was prepared, where two active, non-porous layers with different VPTT were combined. For actuation, the water in the petri dish was first heated to 40 °C and then heated to 60 °C in a second step to sequentially actuate the different VPTTs. The bilayer actuates with three actuation states, but the forward and backward motion are identical (Figure S5).

Preparation and Actuation with Seesaw Geometry

The seesaw geometry was manufactured by filling the porous and non-porous hydrogel precursors in separate rectangular PTFE ($L \times w \times h$ = 4 × 10 × 5 mm) molds followed by photopolymerizing for 60 s with UV light. Afterwards, the two hydrogel blocks were superglued on FFF printed ABS pieces ($L \times w \times h$ = 60 × 15 × 3 mm) to form the moving top of the seesaw. The other side of the hydrogel blocks was then glued to a second FFF printed ABS baseplate ($L \times w \times h$ = 60 × 15 × 3 mm), which was adhered with double sided tape to a petri dish with white foil as background. The seesaw was imaged from above, with the seesaw actuator lying on the side. For actuation, the petri dish was filled with water, which was heated to 60 °C. For re-swelling, ice was added to the water. An image was recorded every 30 s.

Furthermore, seesaws were arrayed with alternating orientation into a conveyer belt, which transports objects. In contrast to the single seesaw, the conveyer belt is standing upright (Figure S7). The conveyer belt was then placed into a 18 L water bath (CC-118A) and a glass marble with 16 mm diameter was placed on top. For better visualization, a white background was placed behind the setup. For actuation, the water was heated to 60 °C to trigger the hydrogel contraction of the conveyer belt. For re-swelling, ice was added to the water to achieve fast cooling to room temperature. An image was recorded every 30 s.

Seesaw Control: A seesaw with active and passive material actuates reciprocally with tilting of the passive bar in one direction (Figure S6). In more detail, the classical seesaw actuator consists of a non-porous actuator and passive material and actuates with two actuation states. FE simulation, which traces the center of the seesaw, reveals the reciprocal movement with zero A_{tr}. The maximum amount of accumulated work is $W_{\max} = 1 * E_{\mathrm{kin}}$.

Seesaw Array Control: A multiresponsive seesaw actuator array was prepared, where two active layers with different VPTT were combined (Figure S8a). For actuation, the water was first heated to 40 °C and then heated to 60 °C in a second step to trigger both VPTT sequentially. The seesaw actuates with three actuation states, where the forward and backward motion are identical (Figure S8b-c). An array of multiresponsive seesaw actuators can transport the sphere only once. Further stimulus application does not result in additional sphere transport.

Structured Illumination Microscopy of the Porous Hydrogel with Porosity Analysis

Structured illumination superresolution microscopy confirms the porosity with a spinodal bicontinuous open-pore structure with porosity of 74 vol%, which describes the interconnected void space of the hydrogel (Figure S1). The porous hydrogel was polymerized onto a round glass bottom petri dish and imaged on a Zeiss Elyra 7 Imaging System using alpha Plan-Apochromat 63x, N.A. 1.46.oil immersion, pco.edge 4.2 CLHS water-cooled sCMOS cameras. For imaging, fluorescein o-acrylate was copolymerized into the hydrogel as dye, which was excited with a λ = 488 nm excitation laser. Due to the opaque nature of the porous hydrogel, total internal reflection fluorescence (TIRF) microscopy with the respective TIRF objective was conducted to visualize the pores of the hydrogel.

The hydrogel porosity was analyzed by calculating the void area from a z-stack (31 stacks of X × Y = 35 × 39 mm^2 with 0.97 µm height) following a literature procedure[5]. Briefly, the z-stack was binarized with the Huang algorithm and the fluorescent polymer network was quantified with the analyze particle function.

Microscale Fabrication via µCOP

The µCOP manufacturing technique was optimized to achieve the multimaterial printing inside a custom-designed microfluidic chip for exchange of hydrogel precursors (Figure S9). The whole printing process requires multiple steps and was inspired by Haris et al.[6] and Rubanov et al.[7].

1. Fabrication of the Master Mold. To obtain desired the dimensions of the microfluid chip, a master mold with positive features was printed with a digital light processing Asiga MAX X27 printer and commercial Moiin Tech Clear resin. To passivate the surface and remove residual monomers that inhibit the curing of polydimethylsiloxane (PDMS) on the mold surface, the surfaces were post-treated with UV and heat, followed by perfluorination[8]. First, the printed master mold was washed 2 – 3 times with ethanol or isopropanol for 2 min each, post-cured under UV ($\lambda_{\max}$ = 365 nm) for 60 min, then placed in an oven for 2 hours at 120 °C. Afterwards, the master mold was plasma treated at 1 mbar for 30 s and placed sideways into a 50 mL falcon tube to which 50 µL of 1*H*,1*H*,2*H*,2*H*-perfluorooctyltriethoxysilane was added. Then static vacuum was applied for 30 min to allow surface deposition and reaction of the silane with the master mold.

2. Fabrication of the PDMS Chip. The perfluorinated master mold was then placed into a petri dish and PDMS (Sylgard 184, 10:1 PDMS to catalyst) was poured over the mold. The petri dish was degassed under vacuum to remove air bubbles and then cured in an oven for 2 hours at 80 °C. The PDMS was cut with a knife and holes for inlet and outlet were punched with a 1 mm biopsy tool to obtain the top part of the mold. To fabricate a closed PDMS channel, the PDMS and glass slide (VWR, $L \times w \times h$ = 76 × 26 × 1 mm) were plasma treated at 1 mbar for 30 s and bonded together by manually pressing followed by heating at 100 °C for 2 min on a hotplate.

3. Multimaterial Printing via µCOP. For printing, a Wintech Lightcrafter 4500 projector with a 405 nm source and output power of 5.5 W was used. As input, 1280 × 800 pixel images were projected, where one pixel corresponds to one mirror (58 µm diameter, diamond orientation) of the DMD device. The projected image was reflected upwards with a cage cube-mounted mirror (CCM1-F01/M, Thorlabs) (Figure S13). In this setup, the measured intensity of the projector inside the chip was 25 mW/cm^2 in the on-state (intensity = 255) and 0.9 mW/cm^2 in the off-state (intensity = 0).

For printing, the previously described porous and non-porous hydrogel precursors were used as inks. To improve printing resolution, the photoinitiator concentration was increased from 3.4 mM to 10.2 mM[9]. The image was focused in z-axis then aligned in the x- and y-axis before adding the inks. The inks were injected with a 5 mL single-use syringe through the chip inlet using microfluidic tubing (outer diameter 0.8 mm). The outlet was connected to a 50 mL waste tube.

The required printing times were calibrated for the porous and non-porous ink with the desired image size beforehand (Figure S14). To this end, the hydrogels were printed with different projection times. Afterwards, the printing time was selected, where the dimensions of the printed structure correspond to the input dimensions.

First, the porous ink was injected and the chip was left to equilibrate for 2 min. Then the projector was turned on, and the image of the first layer (10 pixel × 140 pixel) of a bilayer array was projected for 6 s. After printing of the first layer, the projector was covered to avoid further UV curing. For the second printing step, several milliliters of the second ink were injected until no more schlieren were visible and the chip was left to equilibrate for 5 min. Then, the second image (13 pixel × 140 pixel) was projected for 20.5 s with 3 pixel overlap to ensure polymerization across the layer interface (Figure S15). In the third step, a baseplate was printed with passive pAAm ink for 20 s to connect all bilayers. The hydrogels do not swell between the ink exchange as they are constrained between the PDMS and glass chip. This

facilitates proper alignment of the different materials without distortion. After the three printing steps, water was injected to remove the hydrogel precursor solution.

4. Release of the Multimaterial array. The tri-material hydrogel structure was then released from the PDMS chip by carefully cutting the PDMS off the glass slide with a knife. The hydrogel structure is physically attached to the glass slide, but not to the PDMS due to oxygen diffusion, which inhibits polymerization at the PDMS interface[10]. Inside a water-filled petri dish, the hydrogel structure was carefully removed from the glass with a plastic spatula and left for equilibration over night to remove DMSO.

5. Bonding of the Multimaterial Hydrogel to a Methacrylated Glass Slide. In this step, the printed structure was covalently attached to a new methacrylated glass slide. To this end, a glass slide was washed with water, dried and plasma treated for 30 s at 1 mbar followed by soaking for 1 h in 1 mM 3-(trimethoxysilyl)propyl methacrylate in toluene and rinsing in acetone and water[11]. The methacrylated glass slide was then submerged in water where the hydrogel structure was freely moving. The structure was then carefully aligned and the water was removed to avoid further movement of the hydrogel. Then, passive pAAm ink was added in the petri dish and the baseplate of the hydrogel was overprinted for 20 s to covalently attach the bottom part of the bilayer array to the glass slide. After printing, the glass slide with the attached hydrogel structure was immersed in water over night to remove residual monomer and DMSO.

6. Actuation under Oscillating Temperature. For actuation, an aluminum block with a 15 mL channel looping back onto itself of depth 4 mm, that was milled with a 4 mm tool, was used due to its high heat conductivity (Figure S10). The hydrogel structure, which was attached on the glass slide, was transferred from the previous petri dish by pipetting out the water and placing the glass slide inside the aluminum channel. The aluminum block was placed on a heating plate (ColdPlate, qinstruments) with fast heating and cooling rates of 12 K/min and precise temporal control. A custom Python script was used to set the temperature with defined intervals. The common heating interval for one actuation cycle was 45 °C for 20 min followed by 50 min at 10 °C. This heating-cooling cycle can be repeated multiple times.

7. Flow Tracing via Particle Image Velocimetry. Fluorescent tracer polymer microspheres (0.005 v/v%, 1.00 g/mL, 27-32 µm, UVPMS-BY2, Cospheric) were dispersed in water using surfactants according to the protocol of the manufacturer. The images were acquired with a Nikon SMZ25 fluorescent microscope with a LED ($\lambda_{\text{excitation}}$ = 555 nm) through a quadband filter at a frequency of 1 Hz. The X- and Y-position of approximately 6000 fluorescent particles was determined with a Python script, from which the net displacement was calculated. The script is based on trackpy, a python package[12]. For visualization, the analyzed area was divided into boxes and the net displacement of particles that are transiently present was calculated. This means that the particle displacement was calculated from the point when they enter the coordinates of the specific box until they leave the coordinates. The resulting contour plot gives information about the net displacement of specific locations.

The average net flux was determined by averaging the x-displacement of the particles over the whole area during one actuation cycle of 20 min heating to 45 °C and 50 min of cooling to 10 °C. As some particles were not moving, particles with net x-displacement of less than 20 µm were excluded from the analysis. Furthermore, particles that were traced with less than 25 frames were excluded to reduce random fluctuations.

pH-Responsive Actuation

For the pH-responsive hydrogels, the previously developed porous and non-porous precursors were used and AA was added to obtain porous and non-porous pH-responsive p(NIPAAm-*co*-AA) (Figure S12a). To match the swelling size at the starting conditions, 2 mol% AA was added to the non-porous and 5 mol% AA was added to the porous hydrogel. The hydrogels were photopolymerized as previously described then immersed in pH 2 or pH 12 over night for cyclic switching. The pH-responsive bilayer engine shows non-reciprocal actuation with four states (Figure S12b).

Finite Element Simulations

To predict and optimize the actuation of the soft robotic engines, FE simulations were conducted using the structural mechanics module of COMSOL Multiphysics 6.2. Hydrogels were simulated as incompressible Neo-Hookean materials with a bulk Poisson's ratio of ν_{bulk} = 0.45. All simulations were performed in 2D plane stress settings for specimens. To simulate swelling, experimentally determined actuation strain $\varepsilon_{\text{A}} = 1 - L_{\text{contracted}}/L_{\text{swollen}}$ were used as input for isotropic hygroscopic swelling with $\varepsilon_{\text{swelling}} = \beta * c$ (with β = coefficient of hydroscopic swelling and c = water concentration). To simulate the different swelling speeds due to the kinetic asymmetry, the water concentration c is input as a bounded growth function: $c_{\text{contraction}} = L_{\text{contracted}} - (L_{\text{contracted}} - L_{\text{swollen}}) * \exp\,(-D_{\text{ratio}} * k * x)$. The experimentally determined kinetic asymmetry D_{ratio} is input into the exponent for the faster contracting layer. In the slower contraction layer, D_{ratio} equals 1. For re-swelling, the function is reversed $c_{\text{reswelling}} = L_{\text{swollen}} - (L_{\text{swollen}} - L_{\text{contracted}}) * \exp\,(-D_{\text{ratio}} * k * x)$. This approach assumes homogeneous swelling of each individual layer.

To further optimize the space of experimental parameters for the bilayer, the trajectory, which is the XY position of the tip over time, was determined in **parametric sweeps for bilayer strips**. To quantify the non-reciprocal actuation path, we superimpose the simulated intermediate XY positions and obtain a motion trajectory that allows us to extract the A_{tr} between the forward and backward actuation by monitoring the center of the beam. First, a parametric sweep with varying stiffness ratio E_{ratio} was conducted. Second, the height h of the actuating hydrogel layers were varied at constant width w to determine the dependence of A_{tr} as a function of geometrical dimensions. Third, the actuation strain was varied. Fourth, the kinetic asymmetry D_{ratio} was varied. The results are plotted in Figure S2. When not varied, the following parameters were used: E_{ratio} = 1, D_{ratio} = 20, w_{layer} = 0.7 mm, h = 10 mm, k = 4, ε_{A} = 30 %.

When applying the FE simulation to the seesaw, the center position was determined over time. The following parameters for actuation and geometry were used as input: E_{ratio} = 1, D_{ratio} = 20, w = 5 mm, h = 10 mm, k = 4, ε_{A} = 30 %.

1.2 Supplementary Figures

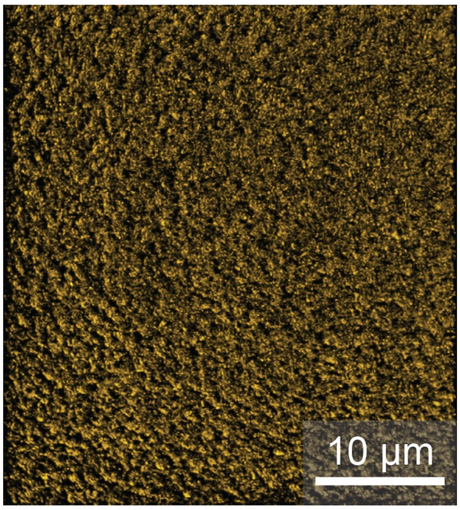

Figure S1. The porosity of the porous (φ_{H2O} = 30 %) pNIPAAm hydrogel visualized by structured illumination superresolution microscopy.

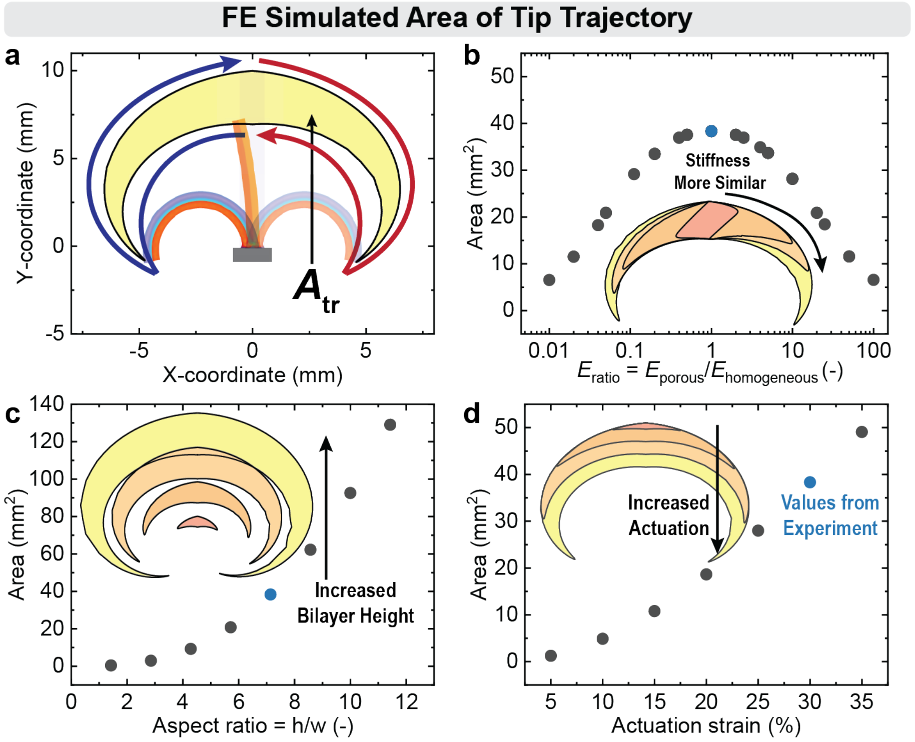

Figure S2. FE simulation of varying parameters from bilayer actuation and varying dimensions. *(**a**) X- and Y-coordinates of the trajectory of the bilayer tip during one heating and cooling cycle. Parametric sweeps of the* A_{tr} *for different (**b**) stiffness ratios of both hydrogel layers, (**c**) bilayer aspect ratios, and (**d**) actuation strain of both hydrogel layers.*

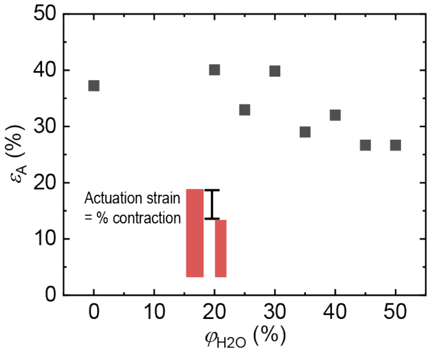

Figure S3. Actuation strain of hydrogels with varying φ_{H2O}***.***

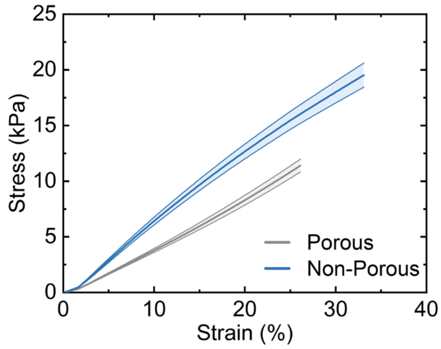

Figure S4. Tensile testing of rectangular porous and non-porous hydrogels. *The graphs represent the averaged curves with standard error of n = 8 specimens (L × w × h = 26 × 5.2 × 2.6 mm³) each.*

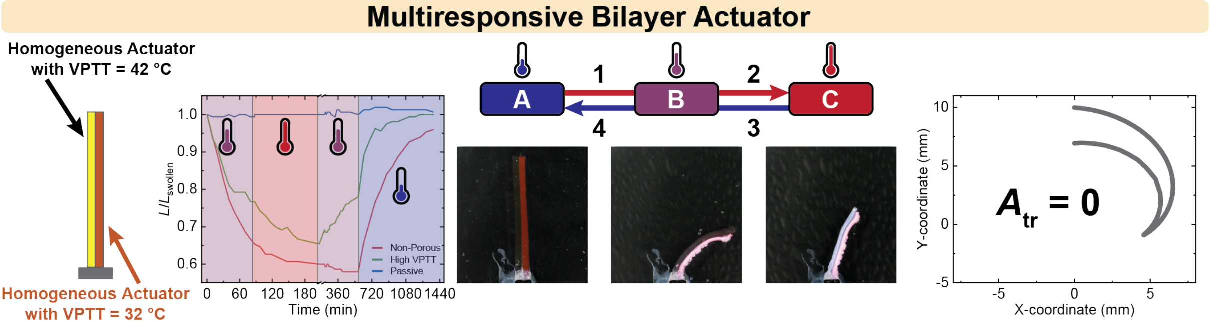

Figure S5. A multiresponsive bilayer actuates reciprocally with three actuation states. *The bilayer is composed of p(NIPAAm-co-AAm) with a VPTT of 42 °C (left) and pNIPAAm with a VPTT of 32 °C (right). Switching was done by heating to 40 °C (state B) and 60 °C (state C) for sequential activation of VPTT. Although bilayer bending is achieved, no work is accumulated. FE simulation reveals that the* A_{tr} *is zero. Heating: Bending goes first to the right (B), then to the center contracted (C). Cooling: Bending goes to the right (B), then to the center expanded (A) on the same motion trajectory.*

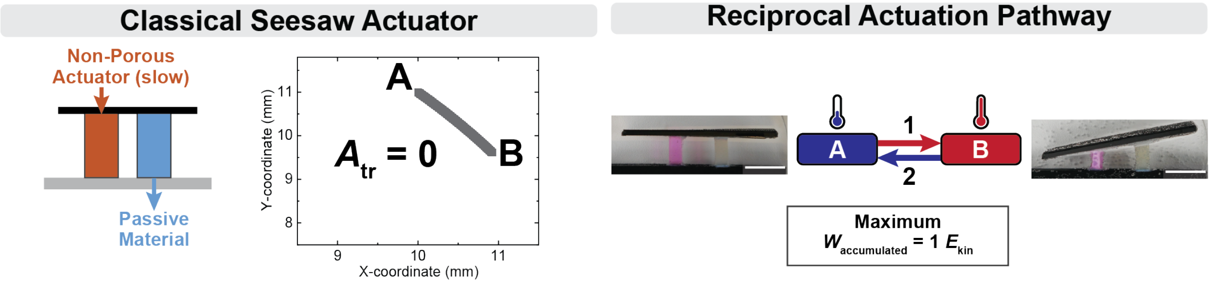

Figure S6. A classical seesaw actuator actuates reciprocally between actuation states A and B. *FE simulations show reciprocal actuation with identical forward and backward motion, resulting in zero* A_{tr}*. The seesaw tilts to the left during heating and returns to its initial state upon cooling.*

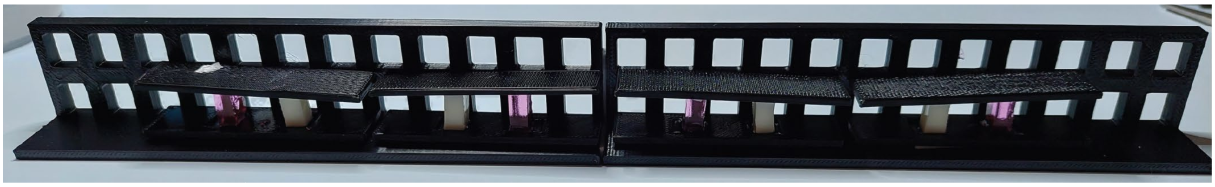

Figure S7. Experimental setup of the conveyer geometry before placing it into the water bath.

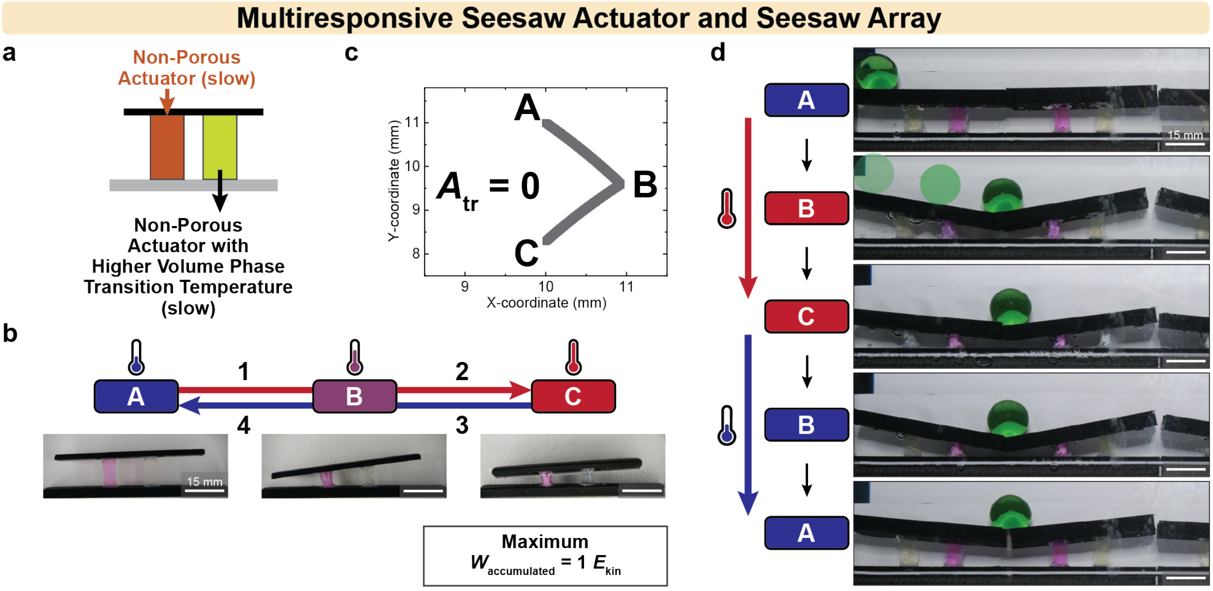

Figure S8. Multiresponsive Seesaw Actuator and Seesaw Array. *(**a**) For comparison, a multiresponsive seesaw actuator with two VPTTs is constructed. The non-porous actuator (2.5 M NIPAAm, 3.7 mol% Bis-AAm) with a VPTT of 32 °C is combined with a second non-porous actuator with a higher VPTT of 42 °C (2.5 M NIPAAm, 0.25 M AAm, 3.7 mol% Bis-AAm in respect to both monomers). (**b**) With two phase transitions, two additional actuation states are obtained. The actuation trajectory is reciprocal. (**c**) FE simulation reveals the reciprocal trajectory of the seesaw center. (**d**) An array of multiresponsive seesaw actuators that are alternatingly flipped move a sphere once during heating. Additional stimulus application cannot further transport the object. No additional work is accumulated during repeated cycles.*

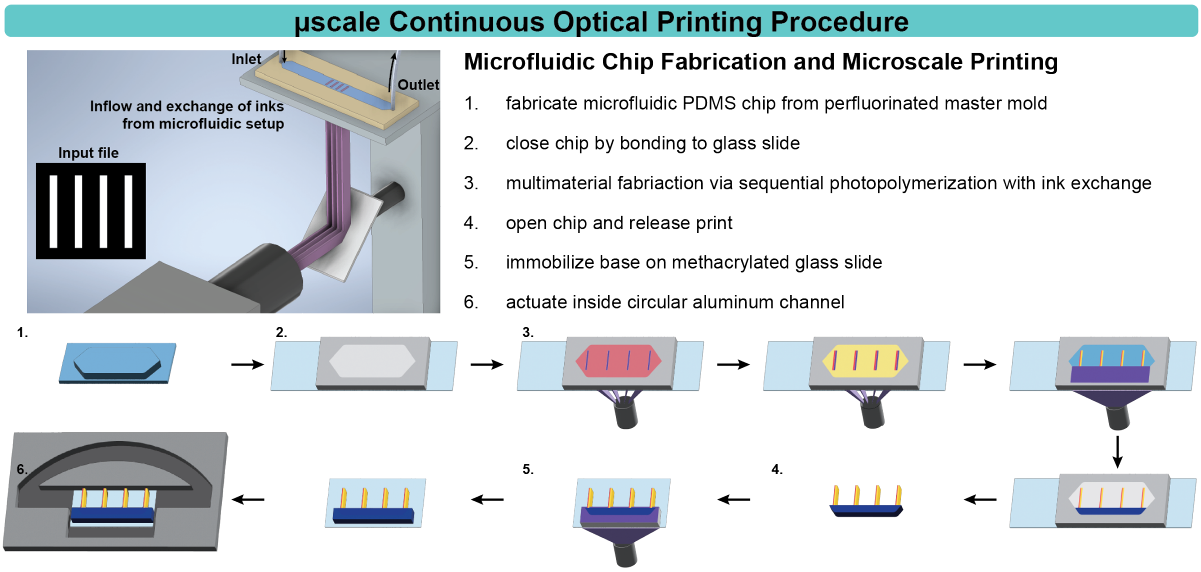

Figure S9. µCOP fabrication procedure with the individual fabrication steps.

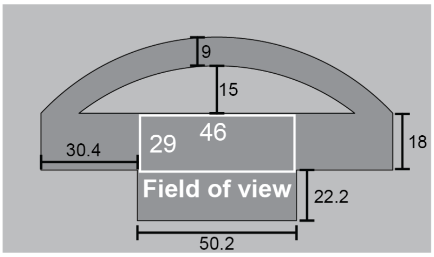

Figure S10. Dimensions of the circular aluminum channel in mm. *The depth is 4 mm.*

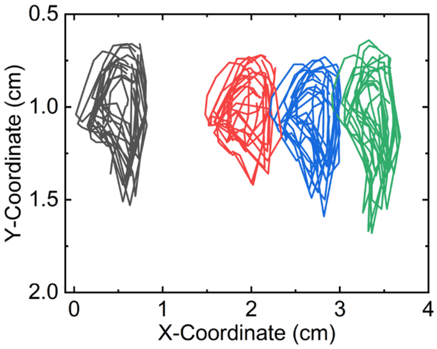

Figure S11. Cycling stability of 4 cilia in an array over 17 cycles of heating and cooling. *The position of the individual cilia tips are traced.*

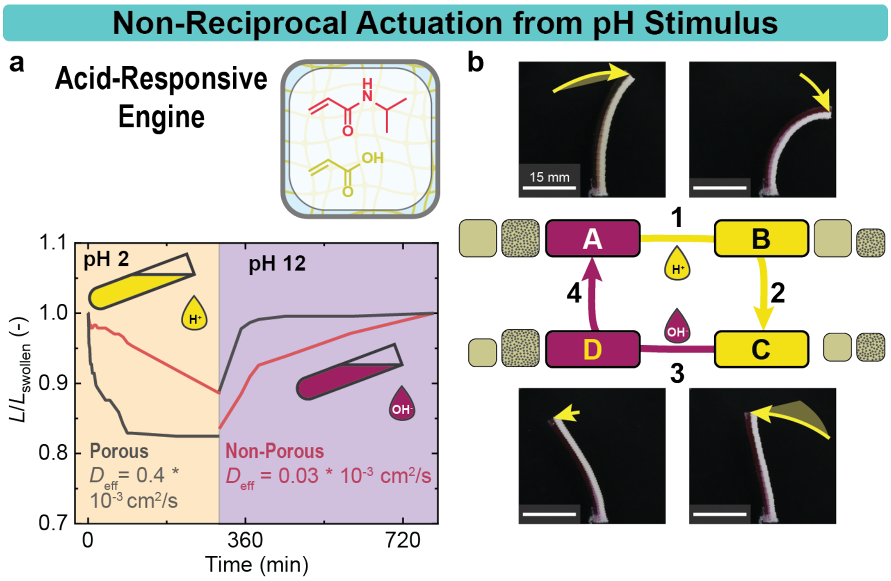

Figure S12. Extension of the concept to pH stimulus. *(**a**) Copolymerizing the thermoresponsive pNIPAAm engine with acrylic acid (AA) renders it pH responsive. Actuation with subsequent low (pH 2) and high pH (pH 12) stimulus reveals kinetic asymmetry in actuation for individual porous and non-porous hydrogels. (**b**) Implementation of the acid-responsive engine into a bilayer geometry results in a non-reciprocal actuation trajectory with four actuation steps during one cycle of stimulus and counter-stimulus application.*

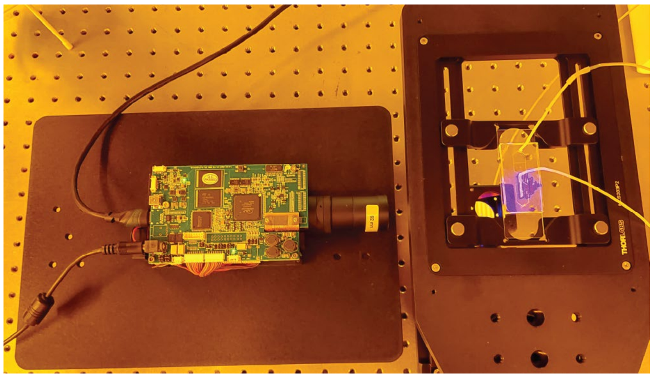

Figure S13. Image of the µCOP setup, which is mounted to an optical table.

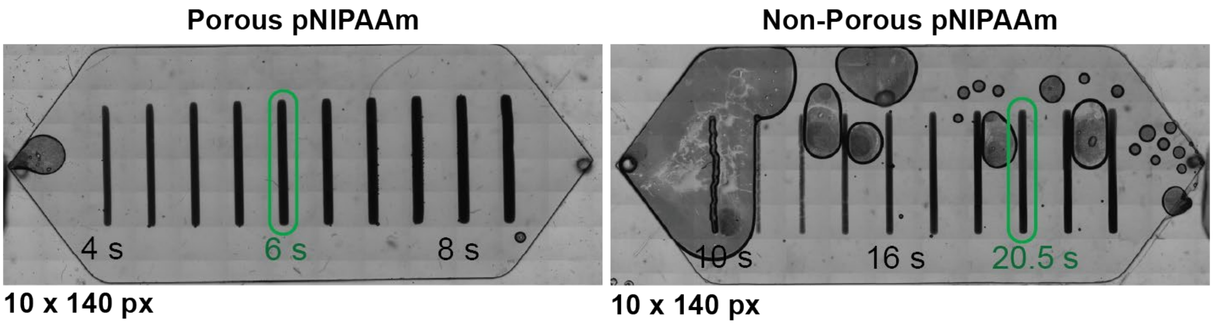

Figure S14. Calibration of the porous and non-porous pNIPAAm ink for a single layer with dimensions 10 pixel × 140 pixel. *The air bubbles appeared after printing and did not interfere with the calibration.*

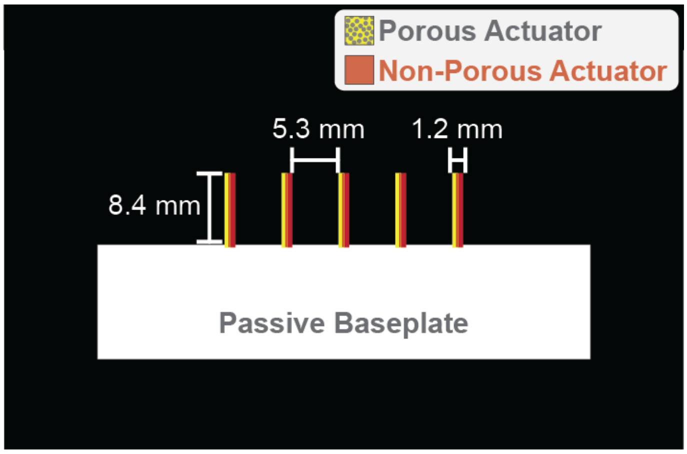

Figure S15. Combined image of the three individual input images, which are here colored differently for better visualization. *The porous layers (yellow) are printed first, followed by the non-porous layers (red) with 3 pixel overlap. Afterwards, a big baseplate with 5 pixel overlap is printed.*

1.3 Supplementary Table

Definitions

Table S1. Definitions of terms used in the main text.

Term	Definition
Motion	**Trajectory of actuation**, which can be reciprocal or non-reciprocal.
Movement	**Net displacement** of fluids or solid objects which **accumulates** with repeated cycles of non-reciprocal motion.
Soft robotic actuator	A soft robotic device that follows a **reciprocal trajectory** and **cannot accumulate work** in continuous operation because forward and backward motion are each other's reverse.
Soft robotic engine	A soft robotic device that follows a **non-reciprocal trajectory** during actuation, where forward and backward motion are different due to kinetic asymmetry in the actuator components. Such devices **accumulate work in cyclic operation** and provide useful macroscopic function such as fluid pumping or object transport.
D_{ratio}	$D_{\mathrm{ratio}} = D_{\mathrm{eff,porous}}/D_{\mathrm{eff,non-porous}}$; the ratio of the effective diffusion coefficient of the porous actuating hydrogel in respect to water as compared to a homogeneous hydrogel. A parameter to quantify kinetic asymmetry in swelling and deswelling kinetics for hydrogels of different porosities.
A_{tr}	The area of trajectory A_{tr} is the area bounded by the trajectory of the tip or center of the moving object during non-reciprocal motion. This serves as a **semi-quantitative parameter for the amount of work done** by an engine and is available by video tracing of differences between forward and backward operation during stimulus/counter stimulus.

1.4 References

[1] H. Tokuyama, N. Ishihara, S. Sakohara, *Polymer Bulletin* **2008**, 61, 399.
[2] T. Tanaka, D. J. Fillmore, *The Journal of Chemical Physics* **1979**, 70, 1214.
[3] Y. Hiei, I. Ohshima, M. Hara, T. Seki, T. Hoshino, Y. Takeoka, *Soft Matter* **2022**, 18, 5204.
[4] Y. Li, T. Tanaka, *The Journal of Chemical Physics* **1990**, 92, 1365.
[5] T. H. Qazi, V. G. Muir, J. A. Burdick, *ACS Biomaterials Science & Engineering* **2022**, 8, 1427.
[6] U. Haris, J. T. Plank, B. Li, Z. A. Page, A. R. Lippert, *ACS Central Science* **2022**, 8, 67.
[7] M. Rubanov, J. Cole, H.-J. Lee, L. G. S. Cordova, Z. Chen, E. Gonzalez, R. Schulman, *PLoS ONE* **2024**, 19, e0295923.
[8] B. Venzac, S. Deng, Z. Mahmoud, A. Lenferink, A. Costa, F. Bray, C. Otto, C. Rolando, S. Le Gac, *Analytical Chemistry* **2021**, 93, 7180.
[9] A. Orth, D. Webber, Y. Zhang, K. L. Sampson, H. W. de Haan, T. Lacelle, R. Lam, D. Solis, S. Dayanandan, T. Waddell, T. Lewis, H. K. Taylor, J. Boisvert, C. Paquet, *Nature Communications* **2023**, 14, 4412.
[10] D. Dendukuri, P. Panda, R. Haghgooie, J. M. Kim, T. A. Hatton, P. S. Doyle, *Macromolecules* **2008**, 41, 8547.
[11] M. Hippler, E. Blasco, J. Qu, M. Tanaka, C. Barner-Kowollik, M. Wegener, M. Bastmeyer, *Nature Communications* **2019**, 10, 232.
[12] D. Allan, T. Caswell, N. Keim, C. van der Wel, R. Verweij, **2024**.
